# Pain in multiple system atrophy and progressive supranuclear palsy compared to Parkinson's disease

**DOI:** 10.1002/brb3.320

**Published:** 2015-03-25

**Authors:** Lewis Kass-Iliyya, Christopher Kobylecki, Kathryn R McDonald, Alexander Gerhard, Monty A Silverdale

**Affiliations:** 1 Department of Neurology, Greater Manchester Neurosciences Centre, Salford Royal NHS Foundation TrustStott Lane, M6 8HD, Salford, U.K; 2 Centre for Clinical and Cognitive Neurosciences, Institute of Brain Behaviour and Mental Health, University of ManchesterManchester, U.K

**Keywords:** Multiple system atrophy, neuropathic, pain, Parkinson's disease, progressive supranuclear palsy

## Abstract

**Background:**

Pain is a common nonmotor symptom in Parkinson's disease (PD). The pathophysiology of pain in PD is not well understood. Pain characteristics have rarely been studied in atypical parkinsonian disorders such as Multiple System Atrophy (MSA) and Progressive Supranuclear Palsy (PSP).

**Aim of the study:**

We aimed to evaluate pain intensity, location, and associated symptoms in atypical parkinsonian disorders compared to PD.

**Methods:**

Twenty-one patients with MSA, 16 patients with PSP, and 65 patients with PD were screened for pain using question 1.9 of the MDS-UPDRS. Pain intensity was quantified using the short form McGill Pain Questionnaire (SFMPQ). Pain locations were documented. Motor disability was measured using UPDRS-III. Affective symptoms were assessed using the Hospital Anxiety and Depression Scale (HADS).

**Results:**

Pain was significantly more common and more severe in PD and MSA compared to PSP (*P *<* *0.01). Pain locations were similar with limb pain being the most common followed by neck and back pain. Pain intensity correlated with HADS scores but not motor severity.

**Conclusions:**

Pain is more common and more intense in PD and MSA than PSP. Differences in distribution of neurodegenerative pathologies may underlie these differential pain profiles.

## Introduction

Pain is an important and common nonmotor symptom in Parkinson's disease (PD) (Beiske et al. 2009). Pain characteristics have been frequently studied in PD, however, there is very little literature addressing pain in atypical parkinsonian disorders. Although there have been studies documenting pain presence in atypical parkinsonian disorders (Colosimo et al. 2010), a prospective comparative study using validated pain measures has never been reported.

Pain in parkinsonian disorders is likely to be mediated by both central and peripheral factors with both neuropathic and nociceptive mechanisms (Wasner and Deuschl 2012). Pain is a very subjective symptom making quantification and characterization a challenge.

Pain research has significantly benefited from the development of scales such as the Short Form McGill Pain Questionnaire (SFMPQ) and the Leeds Assessment of Neuropathic Symptoms and Signs (LANSS) scale, which can, respectively, quantify pain intensity and distinguish pain mechanisms (neuropathic or nociceptive) among patients and observers (Melzack 1987; Bennett 2001). These scales have been used in previous studies to quantify and qualify pain in PD (Negre-Pages et al. 2008; Hanagasi et al. 2011). In this study, we aimed to evaluate pain intensity, location, and associated symptoms in atypical parkinsonian disorders compared to Parkinson's disease using validated pain scales.

## Materials and Methods

Consecutive patients were recruited from movement disorders outpatient clinics at the Greater Manchester Neurosciences Centre. Patients with clinically probable diagnosis of PD, MSA, or PSP according to published criteria and with a mini-mental state examination score of 24 and above were included (Hughes et al. 1992; Litvan et al. 1996; Gilman et al. 2008). Patients with known painful conditions such as neuropathy, radiculopathy, or severe osteoarthritis were not included. Ethical approval was obtained from the Cumbria and Lancashire Research Ethics Committee (Ref. Number 09/H1016/61). All participants gave their informed written consent.

Assessments were carried out in the medication “ON” state. Patients were screened for the presence of pain using question 1.9 of the MDS-UPDRS, which determines the degree of pain or uncomfortable feelings over the past week (0 = no pain, 1 = slight pain, 2 = mild pain, 3 = moderate pain, 4 = severe pain). Scores of 1 and above were considered positive for the presence of pain.

Pain intensity was calculated using the SFMPQ (Melzack 1987). The site of pain was determined by asking patients to locate their pain on a body map. The contribution of neuropathic mechanisms was evaluated using the LANSS (Bennett 2001). The LANSS scale determines the likelihood of neuropathic pain by a series of five questions and a brief sensory examination to detect allodynia and altered pin-prick threshold. A score of ≥ 12 is suggestive of neuropathic pain.

Age, gender, disease duration, use of analgesia, and pain response to dopaminergic therapy were recorded. Motor disability in all patients was assessed using UPDRS-III. Affective symptoms were assessed using the hospital anxiety and depression scale (HADS) (Zigmond and Snaith 1983).

SPSS version 20 (IBM corporation, Armonk, N.Y.) was used to analyze data. Means were compared using Student's *t*-test or one-way ANOVA with post hoc Bonferroni corrections as appropriate. Categorical data were compared using Chi square test. Pearson's correlation was used to test correlation between potentially dependent variables. *P* value of <0.05 was considered significant.

## Results

**Figure 1:**
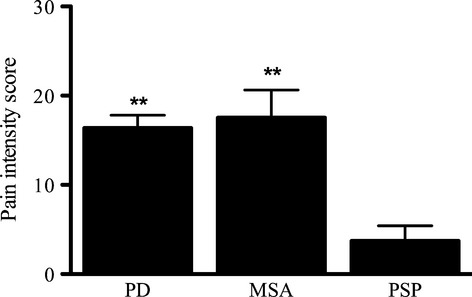
Comparison of pain intensity between parkinsonian disorders as defined by the short-form McGill pain questionnaire. Mean scores and SEM are shown. **P < 0.05 versus PSP.

Sixty-five patients with PD, 21 patients with MSA (14 MSA-P, 7 MSA-C), and 16 patients with PSP were enrolled in the study. Pain was reported in 58 PD patients (89%), 17 MSA patients (81%), and four PSP patients (25%) (*P *<* *0.01). Overall mean pain intensity was significantly greater in MSA and PD compared to PSP (*P < *0.01) (Fig.1). Pain was more common in MSA-P (*n* = 14, 100%) compared to MSA-C (*n* = 3, 43%) (*P *<* *0.01). There was a trend toward higher overall mean pain intensity scores in MSA-P (21.7 ± 3.5) compared to MSA-C (9.3 ± 5.1) (*P* = 0.056).

Of the patients who reported pain, neuropathic pain as assessed by the LANSS scale was found in 19 PD (33%) and three MSA (18%) patients. No patient fulfilled the criteria for neuropathic pain in the PSP group.

Twenty-eight patients with PD (43%) were on regular analgesia with nine patients (15%) taking neuropathic pain treatments (Gabapentin, Pregabalin, or Amitriptyline). Eleven patients with MSA (52%) were on regular analgesia and four patients were taking neuropathic pain treatments (19%). Five patients with PSP were using regular analgesia (31%) and one patient was on amitriptyline. There was no significant statistical difference between these proportions (Table 1).

**Table 1:** Demographic and clinical data on patients with Parkinson's disease, multiple system atrophy, and progressive supranuclear palsy

	PD	MSA	PSP
Number (female)	65 (26)	21 (12)	16 (9)
Age (years)	63.9 (1.2)*	63.6 (1.6)*	73 (1.7)
Disease duration (years)	7.2 (0.6)*$	3.2 (0.3)	3.9 (0.6)
UPDRS-III	22.5 (1.3)*$	37.5 (2.9)	38.1 (2.9)
Pain present	58/65 (89%)*	17/21 (81%)*	4/16 (25%)
SFMPQ pain score	16.4 (1.5)*	17.6 (3.1)*	3.7 (1.7)
Number with neuropathic pain (LANSS score ≥12)	19/58 (33%)	3/17 (18%)	0
Pain improves with dopaminergic therapy	29/58 (50%)	8/17 (47%)	1/4 (25%)
Regular analgesia	28/65 (43%)	11/21 (52%)	5/16 (31%)
Neuropathic pain treatment	9/65 (15%)	4/21 (19%)	1/16 (6%)

Data are presented as mean (SEM) unless otherwise specified. *P <* *0.05 versus PSP; ^$^*P *<* *0.05 versus MSA. UPDRS, Unified Parkinson's Disease Rating Scale; SFMPQ, Short-form McGill Pain Questionnaire; LANSS, Leeds Assessment of Neuropathic Symptoms and Signs; HADS, Hospital Anxiety and Depression Scale.

**Figure 2:**
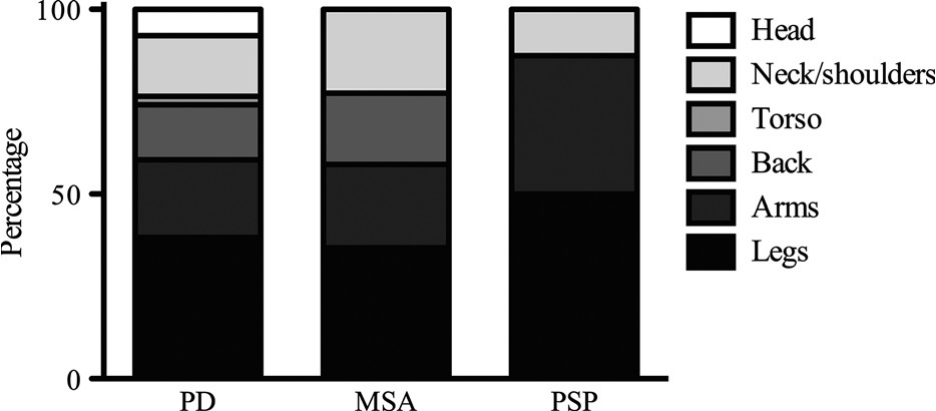
Comparison of pain location between parkinsonian disorders.

The distribution of pain was similar between groups with lower limb pain being the most common followed by upper limb pain, neck pain, and back pain. Bilateral shoulder pain (coat-hanger pain) was similarly prevalent in MSA and PD (Fig.2).

Fifty-one percent of PD patients with pain reported improvement with dopaminergic medications compared to 57% of MSA patients and 25% of PSP patients (Table 2009).

Pain intensity scores correlated significantly with total HADS scores in both PD (*r *=* *0.579, *P *<* *0.01) and MSA (*r *=* *0.734, *P *<* *0.01). There was no correlation between pain intensity and disease duration or UPDRS-III scores.

## Discussion

Our main finding is that pain in PD and MSA is significantly more intense and prevalent compared to PSP. Pain is also more burdensome in MSA-P than MSA-C.

Despite the high prevalence of pain in PD and MSA only half of these patients were taking regular analgesia indicating that pain related to parkinsonian syndromes might be under-recognized and under-treated in these conditions.

Different anatomical patterns of neurodegeneration affecting pain pathways as well as different pathological substrates of synucleinopathies versus tauopathies may explain this discrepancy in pain profiles. Neurodegeneration affecting the basal ganglia would be expected to alter pain perception given the involvement of these structures in pain processing (Borsook et al. 2010). The greater involvement of the basal ganglia in MSA-P compared to MSA-C could account for the observed difference in pain prevalence. Cognitive factors in PSP could potentially reduce pain perception (Carlino et al. 2010). Patients with frank dementia were excluded, however, more sensitive tests of frontal lobe function were not performed.

Neck and shoulder pain (coat-hanger pain) have been considered to be a feature of MSA. We found neck and shoulder pain to be similarly prevalent in PD. The results suggest that coat-hanger pain may not necessarily constitute a “red flag” for MSA.

When assessed using the LANSS scale a small proportion of patients' pain had probable neuropathic mechanisms. This is also supported by the number of patients who were taking regular neuropathic pain treatments (Table 2009). Although no comprehensive assessments were made to distinguish neuropathic pain from nociceptive pain it is likely that central mechanisms play at least a partial role in the generation of pain in parkinsonian disorders. Furthermore, reduced threshold to experimental pain is reported in all three conditions suggesting involvement of central mechanisms (Brefel-Courbon et al. 2005; Stamelou et al. 2012; Perrotta et al. 2013).

Over half of our patients with PD and MSA reported improvement of their pain with dopaminergic medications. Thus, optimizing dopaminergic treatment may be important in MSA for the management of nonmotor symptoms such as pain even when the motor symptoms are poorly responsive to treatment.

The strong correlations between pain intensity and affective symptoms may reflect common pathophysiological substrates or a secondary effect of pain. This has been documented in previous reports investigating pain in PD (Negre-Pages et al. 2008). Depression is equally common in both PSP and MSA and has been found to have a significant influence on patients' perception of their quality of life (Schrag et al. 2010). This highlights the importance of adequate management of affective symptoms for pain control and improved subjective health status.

There are a number of limitations to our study, the main one being the relatively small sample size. This is largely due to the relative rarity of atypical parkinsonian disorders and the exclusion of patients with dementia, limiting the number of suitable PSP patients. Motor disability was not evenly matched between the three groups, as atypical parkinsonian syndromes are more aggressive conferring higher motor scores and PD patients were assessed in the “ON” state in an outpatient setting. Assessing PD patients in the “OFF” state would have probably been more reflective of the degree of motor impairment. This was difficult given the cross-sectional design of the study, which was conducted in an outpatient setting. However, contrary to what one would expect, pain intensity in PSP and MSA was either less or matching pain intensity in PD despite higher motor disability. Therefore, the discrepancy in motor scores is unlikely to have contributed to the difference in reported pain. Disease duration and age were also different between the groups reflecting the natural disease course and age groups at which these conditions present. Due to sample size we could not correct for these variables and we accept this as another limitation to our study.

To our knowledge, this is the first study to prospectively evaluate pain in atypical parkinsonian disorders using validated pain scales. Further work with larger cohort and multiple regression analysis is needed to understand the causes of pain in parkinsonian disorders and alleviate the burden of this common and disabling symptom.
